# Preliminary evidence of different selection pressures on cancer cells as compared to normal tissues

**DOI:** 10.1186/1742-4682-9-44

**Published:** 2012-11-12

**Authors:** Katie Ovens, Christopher Naugler

**Affiliations:** 1 Bachelor of Health Sciences Program, Faculty of Medicine, Room G503, O’Brien Centre for the BHSc, 3330 Hospital Drive NW, Calgary, AB, T2N 4N1, Canada; 2 Calgary Laboratory Services and Department of Pathology and Laboratory Medicine, University of Calgary, C414, Diagnostic and Scientific Centre, 9, 3535 Research Road NW, Calgary, AB, T2L 2K8, Canada

## Abstract

**Background:**

Cancer is characterized by both a high mutation rate as well as high rates of cell division and cell death. We postulate that these conditions will result in the eventual mutational inactivation of genes not essential to the survival of the cancer cell, while mutations in essential genes will be eliminated by natural selection leaving molecular signatures of selection in genes required for survival and reproduction. By looking for signatures of natural selection in the genomes of cancer cells, it should therefore be possible to determine which genes have been essential for the development of a particular cancer.

**Methods:**

We provide a proof of principle test of this idea by applying a test of neutrality (Nei-Gojobori Z-test of selection) to 139 cancer-related nucleotide sequences obtained from GenBank representing 46 cancer-derived genes.

**Results:**

Among cancer associated sequences, 10 genes showed molecular evidence of selection. Of these 10 genes, four showed molecular evidence of selection in non-cancer transcripts. Among non-cancer associated sequences, eight genes showed molecular evidence of selection, with four of these also showing selection in the cancer associated sequences.

**Conclusions:**

These results provide preliminary evidence that the same genes may experience different selection pressures within normal and cancer tissues. Application of this technique could identify genes under unique selection pressure in cancer tissues and thereby indicate possible targets for therapeutic intervention.

## Introduction

Cancer cell clones evolve over the lifespan a tumour [1-3]. The selective pressures driving this clonal evolution are myriad and may include microenvironmental factors, immune system surveillance, competition with other cancer and somatic cells, and selective killing of cancer cells by surgery, chemotherapy and radiation [2-9]. Two features of cancer portend intense natural selection among cancer cells. The first is the observation that cancer cells (at least in the later stages of growth) experience a high rate of cell death [10]. The second is the greatly increased rate of mutations in cancer cells [11-16]. For example, a recent large scale study identified mutations in 11% of protein coding genes examined over 756 cancer cell lines [17]. Many of these mutations, even if they change the resulting protein sequence of the gene product may be considered to be “passenger” mutations that do not contribute to oncogenesis [16] and are of no significance to the cancer cell [3,12]. Indeed mutations in non-essential genes may even be adaptive to the cancer cell as they shed costly metabolic processes irrelevant to reproduction of the cancer cell [3].

The high mutation rate and rapid cellular turnover may be expected to form an intense environment for natural selection where mutations arise and are tested for functional importance through competition with other cells. Eventually, this environment may lead to the situation where many genes have been rendered nonfunctional by mutations and the subset of genes that have been important for the survival and multiplication of the cancer cells will have been preserved through constant selection of functional versions of these genes.

Evolutionary biologists have identified a number of methods for detecting molecular evidence of natural selection [18]. These, so-called “tests of selection” attempt to differentiate neutral evolution (i.e. genetic drift) from Darwinian selection. One commonly used method compares ratios of synonymous and non-synonymous base substitutions. This approach has the advantage of being robust with regards to population growth [18], a confounding factor particularly important in the context of cancer cell growth. Synonymous base substitutions change the exonic base pair sequence but conserve the translated amino acid sequence (because of the degenerate nature of the DNA code). In contrast, nonsynonymous base pair substitutions change both the base pair sequence as well as the translated amino acid sequence. An increased rate of synonymous to nonsynonymous base substitutions provides evidence that the base sequence in question is or has been under natural selection to conserve the amino acid sequence (purifying selection). Less commonly, a sequence may exhibit an increased rate of nonsynonymous to synonymous base substitutions, indicating the base sequence in question has been under natural selection to change the ancestral amino acid sequence (diversifying selection). Perhaps the best described example of this is the diversifying selection shaping the peptide binding grooves of MHC class I molecules [19]. We might expect that the majority of selection pressures on cancer cells would be in the form of purifying selection to maintain the function of essential genes. However it is also possible that diversifying selection also plays a role in cancer cell evolution, possibly in facilitating the exploitation of new microenvironments.

Here we test the hypothesis that due to the high mutation rates and increased cell turnover in cancer cells, genes of importance to the survival of the cancer cell should show molecular evidence of natural selection. Furthermore, we predict that in the majority of cases this selection would be in the form of purifying selection.

## Materials and methods

As an initial test of this hypothesis we obtained cancer-derived DNA sequences from GenBank using the search parameters “carcinoma expression library", "cancer-associated transcript”, "tumour-associated transcript" and “*Homo sapiens”*. We did not attempt to obtain an exhaustive list of all available transcripts but rather sought a convenience sample of different genes where at least two different examples of the same gene sequence from cancer tissue could be obtained. We did not include animal model-derived sequences or experimental cell line sequences. To determine if these genes show natural selection in non-cancerous tissues, Genbank was again used to find non-cancer versions of the same genes. In cases where we could not locate two non-cancer sequences from among the GenBank entries, we isolated the relevant sequences from the NCBI reference sequences primary and alternate assemblies. The sequences used in this study are all publically available from NCBI; the sequence references are given in Table 1.

**Table 1:** Gene sequences used in the analyses

**Gene**	**GenBank Accession Numbers (cancer-related sequences)**	**Probability of Null hypothesis (Hs=Hn) in cancer-related sequences**	**Type of selection**	**P-distance**	**GenBank Accession Numbers (non- cancer-related sequences)**	**Probability of Null hypothesis (Hs=Hn) in non-cancer-related sequences**	**Type of selection**	**Gene function**
EGFR	GI:998566 GI:998564	0.603	none	0.290	GI:229892268 GI:229892301 GI:229892299	0.212	none	Growth factor receptor
LNCaP	GI:429094 GI:429093 GI:429091	0.771	none	0.550	GI:19924155 GI:19924154	1.000	none	protease present in seminal plasma
HYAL1	GI:24497567 GI:24497563 GI:24497560	0.088	none	0.054	GI:386365498 GI:385648248 GI:385648249	0.751	none	candidate tumor suppressor locus
ALDOA	GI:15488980 GI:15277570 GI:38197497	0.234	none	0.090	GI:342187210 GI:342187198 GI:342187192	0.331	none	glycolytic enzyme
PTPN3	GI:223941890 GI:223941884 GI:223941878	0.214	none	0.037	GI:223941893 GI:223941875	0.081	none	protein tyrosine phosphatase
NBL1	GI:323462168 GI:323276671 GI:323462166 GI:323462167	0.794	none	0.045	NC_000001.10 AC_000133.1	0.549	none	bone morphogenetic protein antagonist
BLCAP	GI:47939094 GI:28839694	0.385	none	0.033	GI:268370219 GI:268370215 GI:268370223 GI:268370217	0.077	none	tumour suppression gene
PRKA	GI:331284157 GI:116174749 GI:331284159	0.077	none	0.000	GI:331284154 GI:331284152 GI:331284159	0.497	none	regulates the effect of the cAMP-dependent protein kinase signaling pathway
STEAP2	GI:350276262 GI:350276256 GI:350276260	0.301	none	0.128	GI:100913195 GI:100913193 GI:100913197	0.484	none	metalloreductase
MAGED2	GI:41350319 GI:29171703 GI:29171704	0.899	none	0.015	NC_000023.10 AC_000155.1	0.034	diversifying	negative regulator of wild type p53 activity
FOLR1	GI:262331568 GI:262331573 GI:262331571 GI:262331569	0.470	none	0.007	NC_000011.9 AC_000143.1	1.000	none	folate receptor
EWSR1	GI:48734726 GI:38197249 GI:15029674 GI:13435962	0.310	none	0.072	GI:253970505 GI:253970501 GI:253970497 GI:253970503	0.171	none	involved gene expression, cell signaling, and RNA processing and transport
SHARPIN	GI:21706472 GI:19264111	0.451	none	0.003	GI:333805638 GI:118918414	0.290	none	Involved in NF-kappa-B activation and regulation of inflammation
NUDCD1	GI:13111833 GI:27694435 GI:21411491	0.806	none	0.005	GI:189571676 GI:189571678	0.071	none	tumor-associated antigen
PMEPA1	GI:51593770 GI:16198474 GI:22121998 GI:9255808	0.878	none	0.032	GI:40317615 GI:40317617 GI:40317619	0.353	none	Involved in down-regulation of the androgen receptor
CTAGE5	GI:313882513 GI:30411006 GI:39963693 GI:24659234	0.244	none	0.007	GI:134053863 GI:134053924 GI:134053890	0.002	purifying	tumor-associated antigen
IRAK3	GI:34785939 GI:46854383	0.791	none	0.003	GI:216547518 GI:216547503	1.000	none	receptor-associated kinase
PLS3	GI:34785158 GI:25058020 GI:288915540	0.255	none	0.015	GI:288915537 GI:288915538	0.243	none	actin-bundling protein
PRAME	GI:33874094 GI:25123208 GI:21328745	0.121	none	0.019	GI:46249372 GI:46249366 GI:46249370 GI:46249365	0.754	none	transcriptional repressor
TNFSF13	GI:33873809 GI:24934971 GI:211938417	0.211	none	0.162	GI:211938416 GI:310750386 GI:310750384	1.000	none	possibly involved in regulation of tumor cell growth and monocyte/macrophage-mediated immunological processes
ENOX2	GI:17939422 GI:80478559	0.070	none	0.006	GI:32528292 GI:32528290	1.000	none	growth-related cell surface protein
UQCC	GI:114108213 GI:77415336 GI:111598967 GI:296923772	0.452	none	0.008	GI:296923778 GI:296923775	1.000	none	enzyme involved in ubiquinol-cytochrome c reductase complex
ALOX15B	GI:85067502 GI:85067498 GI:39645887 GI:85067500	0.085	none	0.001	GI:182765463 GI:260166611	1.000	none	involved in the production of fatty acid hydroperoxides
TACSTD2	GI:166795235 GI:238914823 GI:14495610	0.340	none	0.014	NM_002353 AC_000133	1.000	none	cell surface receptor that transduces calcium signals
RAET1E	GI:149790140 GI:73909189	0.197	none	0.151	GI:21040248 GI:341915375 GI:343183384	0.586	none	delivers activating signals to NK cells
MAGEB1	GI:15489350 GI:164693182 GI:49456482	0.337	none	0.011	GI:284004909 GI:257796251 GI:257796250	0.0151	purifying	tumor-associated antigen
CSF1	GI:18088910 GI:166235151	0.413	none	0.142	GI:347360911 GI:166235149 GI:384475524	0.657	none	cytokine that controls the production, differentiation, and function of macrophages
CKAP2	GI:148664243 GI:148664200 GI:187950332 GI:15012012	0.278	none	0.084	NC_000013.10 AC_000145.1	0.320	none	involved in regulating aneuploidy, cell cycling, and cell death
MUC1	GI:182252 GI:115528448 GI:324120948	0.085	none	0.389	GI:324120957 GI:324120955 GI:324120951 GI:324120950	0.716	none	adhesion and anti-adhesion protein; involved in cell signaling
TMPRSS3	GI:14709533 GI:33991397 GI:145701031	0.736	none	0.008	GI:291167774 GI:145701029 GI:291167776	1.000	none	serine protease; plays a role in hearing
FOLR2	GI:34785969 GI:166064049 GI:166064051	0.090	none	0.005	GI:166064049 GI:166064053 GI:166064055	1.000	none	folate receptor
BYF3	GI:33873803 GI:110624585 GI:83641884 GI:83641883	0.137	none	0.049	GI:224177471 GI:1435190 GI:179571 GI:179575	0.686	none	involved in transcriptional initiation
TPBG	GI:33872201 GI:262205658 GI:262205664	0.807	none	0.012	NC_000006.11 AC_000138.1	0.521	none	possible cell adhesion molecule
EMP2	GI:16307197 GI:64692933	0.298	none	0.002	NC_000016.9 AC_000148.1	1.000	none	epithelial membrane protein
TGM6	GI:33331029 GI:33331031	1.00	none	0.000	NC_000020.10 AC_000152.1	0.266	none	associated with central nervous system development and motor function
NBR1	GI:112382227 GI:112382229 GI:112382228	0.359	none	0.504	GI:33869357 GI:111120332	0.018	purifying	Function unknown
CALM2	GI:19913528 GI:16924228 GI:14250064	0.04	diversifying	0.524	GI:229577210 GI:13097164	0.026	diversifying	Mediates enzymes, ion channels and other proteins
NRG1	GI:49522882 GI:34782767 GI:33873543	0.012	purifying	0.220	GI:236460384 GI:236464355	1	none	signaling protein that mediates cell-cell interactions
SYTL2	GI:244790015 GI:244790004 GI:244790009 GI:244790019	0.012	purifying	0.097	GI:82571721 GI:34784984 GI:21951814 GI:21984183	0.482	none	Involved in vesicle trafficking and melanosome distribution
BCAP31	GI:213511729 GI:213511011 GI:213511507 GI:40807164 GI:15680022	0.048	purifying	0.055	GI:374253795 GI:374253793	0.083	none	multi-pass endoplasmic reticulum transmembrane protein
ILK	GI:3150001 GI:16306740 GI: 8648884	0.013	purifying	0.007	GI:62420874 GI:62420871 GI:62420872 GI:8308037	0.222	none	serine/threonine protein kinase
LIMS1	GI:13529136 GI:336455030	0.016	purifying	0.007	GI:164697166 GI:34528462 GI:336455029	<0.001	purifying	likely involved in integrin signaling
CHST4	GI:23273964 GI:262205557 GI:262205902	0.016	purifying	0.021	GI:262205902 GI:262205557	0.018	purifying	sulfotransferase; modifies glycan structures on ligands of the lymphocyte homing receptor L-selectin
EBAG9	GI:17389375 GI:13528905 GI:18490914	0.009	purifying	0.072	GI37694064 GI:37694063 GI:158254733	0.289	none	tumor-associated antigen
NACA	GI:76779232 GI:333033786 GI:163965363	0.002	purifying	0.186	GI:85397251 GI:85397957 GI:60116922	0.755	none	Component of nascent polypeptide-associated complex; prevents mistranslocation of proteins
OCIAD1	GI:13097314 GI:56789926	0.036	purifying	0.065	GI:269914125 GI:269914123 GI:269914126 GI:269914124 GI:269954665	0.005	purifying	tumor-associated antigen

P-distances are given for cancer-associated sequences only. See text for further explanation.

Analyses were performed using the Molecular Evolutionary Genetics Analysis (MEGA) software Version 5 [20]. Following sequence alignment using the ClustalW method, the Nei-Gojobori Z-Test of Selection [21] was used to calculate the synonymous to nonsynonymous base substitution rates and the associated statistical probabilities. P-values of less than 0.05 were considered significant.

## Results

A total of 46 cancer-derived genes represented by 139 sequences were identified (Table 1). No sequences were derived from propagated cell lines. However, we were unable to determine what proportion of examples were from primary tumors vs metastatic tumors. Of the 46 genes, nine genes showed evidence of purifying selection and 1 showed evidence of diversifying selection (Table 1). Six genes showed molecular evidence of selection only in cancer associated sequences (all in the form of purifying selection), four genes showed evidence of selection only in non-cancer associated sequences (three cases of purifying selection and one case of diversifying selection), and finally four genes showed molecular evidence of selection in both cancer and non-cancer associated sequences (three cases of purifying selection and one case of diversifying selection; Table 1). Table 1 also gives the GenBank accession numbers for all sequences used as well as sequence divergence estimates (p-distances) and the results of the Nei-Gojobori Z-tests of selection.

If signatures of selection become more common as mutations accumulate in a cancer-associated sequence, we might expect to see greater nucleotide divergence estimates in examples showing significant selection. To test this, we compared p-distances in the 10 examples showing molecular evidence of selection in the cancer associated sequences with the 36 examples not showing evidence of selection in the cancer associated sequences. The mean p-distance of sequences showing evidence of selection was 0.125, while the mean p-distance of sequences not showing evidence of selection was 0.082 (unpaired *t*-test, p=0.398).

## Discussion

We describe a proof of principle test of a method of identifying molecular signatures of natural selection in cancer-derived gene sequences. We also show that in a sample of 46 genes the cancer and non-cancer derived sequences show different patterns of selection.

As a cancer grows and evolves and different genes come under selection pressure, natural selection may be expected to record evidence of this selection in the proportion of synonymous to nonsynonymous base substitutions as we have discussed here. Even if that particular gene later becomes non-functional through further mutations, evidence of prior selection pressure would be expected to persist. Thus a list of genes showing molecular evidence of selection only in cancer cells could be considered to be those genes which have been important to the survival of the cancer cell up to that point on time. In essence, this provides us with a method to determine which genes have been integral to the survival the cancer cell.

There are several potential weaknesses to our study. First, a different number of sequences were available for the various genes we examined. With a greater number of sequences we may expect a greater power to detect signatures of selection. To test such an effect we compared the mean number of sequences from genes which showed selection (3.17) to the mean number of sequences from genes which did not show selection (3.27). The difference was not statistically significant (p=0.134, unpaired *t*-test). Therefore, although this is a potential theoretical concern, we can find no evidence of this in our data.

Second, we do not have information about the geographic or racial origins of the individuals from whom the cancer and non-cancer gene sequences were derived. It is possible that increased variability noted for some genes could be due to these factors.

Third and perhaps most importantly, the choice of the model to calculate dN/dS as well as the test interpretation are both potentially controversial. The Nei-Gojobori method is perhaps less conservative than a maximum likelihood model but at the same time if the majority of sites in a protein evolve under purifying selection (as we might expect in a functionally essential gene in a tumour) the dN/dS statistic has reduced sensitivity to detect positive selection [22]. Moreover, the behaviour of dN/dS statistics when applied to polymorphisms within a population may behave differently than when applied to fixed mutations between species [23]. Whether cancer cells from the same tumour and/or from tumours from different individuals are sufficiently diverged to be considered analogous to different species [24] is a critical unanswered question. Therefore, because of these uncertainties, we decided to use the simple Nei-Gojobori statistic for this preliminary analysis. As major cancer sequencing initiatives begin producing whole genome sequences from paired cancer/normal samples from the same patient, this question will become more important. Further work should critically examine the optimal statistic to be used for these analyses.

Although we could not detect a statistically significant difference in the mean p-distances between cancer associated sequences showing evidence of selection and those that did not, there was a trend toward greater p-distances among the sequences showing selection and so our inability to demonstrate a difference may be a factor of the limited sample size.

Parenthetically, the process postulated here, where relentless mutation in cancer cells results in either mutational inactivation of genes or positive selection to maintain their function gives a functional explanation for why more advanced cancers invariably show what pathologists refer to as “de-differentiation”; as Mueller’s ratchet [25] removes all but the reproductively essential genes.

It will be obvious that the ability of gene sequences to display evidence of natural selection is based both on a high cancer cell mutation rate and an increased cancer cell proliferative rate which together provide the raw material on which selection can act. As these conditions likely are greater in more advanced cancers, we would expect to see greater molecular evidence of selection in later stage cancer cells. Indeed, comparison of early and later stage cancer cells could provide a roadmap of when particular genes experience selection pressure and therefore when these genes are important for tumorigenesis. Furthermore, because the molecular signatures of selection would be expected to persist for many generations of cancer cells, late stage cancers would be expected to contain a molecular record of genes conserved at essentially any stage of the clonal evolution of the cancer cell, even if that gene is no longer under selection pressure or even is no longer functional. By this line of reasoning, genes which are epigenetically silenced would be shielded from selection and may be expected to eventually be subject to loss of function mutations, even if they maintain molecular evidence of prior natural selection during tumorigenesis.

We caution that our results with regards to specific genes should be interpreted as preliminary only. Our sample was based only on publicly available sequences and encompassed a number of different malignancies making any conclusions about gene function based on these findings premature. Furthermore, this approach may not distinguish between driver genes which promote oncogenesis and non-driver genes nevertheless essential for cancer cell growth and reproduction. However, the application of previously described methods could be used to distinguish these [16,17].

As new databases of cancer genomes become available [14,17-27], a future direction for this work will be to apply these techniques to whole genome sequences of cancer cells. This could be performed at the level of the tumour as a whole to look at genes important across a sample of tumours of the same type or it could be applied to single cells to explore the genes of importance in particular microenvironments such as metastatic deposits. This approach, combined with oncogenetic reconstruction of cancer clonal lineages using the same sequencing data could provide a powerful new tool to identify candidate genes of functional significance for potential targeted therapies as well as providing new insights into the evolutionary mechanisms of cancer cell clonal evolution.

## Conclusions

Genes may be under different selection pressures within a cancer as compared to normal tissues. In this paper we proposed a method to answer the question of what genes are important to a cancer cell. The high mutation rates and rapid cell division present in cancer suggests that functionally important genes will show evidence of selection. We could therefore, in an indirect manner, observe what genes a cancer cell needs to survive. The genes that are important could then form a list of possible targets for therapeutic intervention.

## Competing interests

The authors declare that they have no competing interests.

## Authors’ contributions

KO participated in study design, performed the majority of data analysis and drafted the manuscript. CN conceived of the initial study design, critically revised the manuscript and performed some of the data analyses. All authors read and approved the final manuscript.
